# The Surgery of the Intestines

**Published:** 1906-01-13

**Authors:** 


### PROGRESS IN MEDICINE AND SURGERY.

### THE SURGERY OF THE INTESTINES.

**Intestinal Obstruction** is one of the most difficult problems with which the surgeon has to deal. Barnard,[1] in discussing its general pathology, says it is a composite condition in the production of the symptoms of which three factors participate in variable proportions: (*a*) Obstruction of the lumen of the gut; (*b*) obstruction of the vessels of the gut, but more especially of the veins; (*c*) interference with the nerves and muscular walls of the gut. In one great class of cases of obstruction the lumen of the gut is alone involved, and the vessels and nerves are not interfered with. To this class belong strictures, foreign bodies, chronic intussusception, and obstruction due to the pressure on the gut from without. It will be seen that nearly all such cases are examples of chronic intestinal obstruction. When the vessels are not obstructed the gut is not paralysed, and obstruction of the lumen is in nearly all cases partial only. This condition and the symptoms to which it gives rise are very similar to those of an incarcerated hernia. In the second great class of cases the blood-vessels, but more especially the veins, are obstructed in addition to the lumen of the gut. In this class are found bands, apertures, internal herniæ, acute intussusceptions, and volvulus. These are all examples of acute obstruction. The obstruction in these cases is complete and absolute, for not only do these causes much more effectually close the lumen of the gut, but the interference with the vessels paralyses its wall. The conditions in this class present a very close resemblance to strangulated hernia. Finally, the gut may be paralysed and obstructed by interference with its nerves and muscular coats; and under this heading must be grouped cases of acute peritonitis and acute pancreatitis and perinephric inflammation in which the nerves to various parts of the colon are so interfered with that the contents accumulate in and distend the portions of the colon above them. The occurrence of fæcal vomiting in case of intestinal obstruction is thus explained by Barnard: The contents of the intestines accumulate above the obstruction and by their decomposition irritate the intestinal walls to a profuse secretion which adds enormously to their bulk. As a consequence of this, fresh coils of intestine above are filled with the irritating fluid and provide a fresh source for further secretion; however, the upper part of the alimentary canal is chiefly secretory and the lower absorbent—at least as regards fluid. The secretion of saliva, for instance, is two or three pints a day, and probably the gastric, biliary, and pancreatic secretions are each not much less. The chief areas of absorption are the lower ileum and especially the colon. When a block is interposed between the region of secretion and that of absorption, the gut above must be rapidly filled up by the mixed secretion poured into it from the upper portion of the alimentary canal. When the stomach is invaded by the rising flood, it rejects the surplus as it accumulates, and this accounts for the vast quantity and continuous character of the vomiting. This view—that the major part of the vomited matter is supplied by the salivary, gastric, biliary, and pancreatic secretions—is borne out by the following considerations: Vomiting is more profuse and urgent, and earlier becomes "fæcal," the higher up the alimentary canal the obstruction occurs. When a large gall-stone enters the duodenum by ulceration from the gall-bladder the vomiting is stupendous. As the stone passes down the alimentary canal the vomiting remits and only recurs when the stone finally closes the lumen of the narrow lower ileum. When the obstruction is in the colon, so that a part of the absorptive area is available above it, the vomiting occurs late in the attack and is seldom fæcal or urgent. The differential diagnosis of intestinal obstruction is sometimes a very complex and difficult matter, and, from the point of view of immediate operation, it requires to be distinguished from the following conditions: 1. An organic obstruction is present, but for purposes of treatment it is not intestinal obstruction. Such conditions are hernia and fæcal accumulation. 2. The symptoms are due to peritonitis or irritation of the peritoneum, such as occurs in some cases of appendicitis, and tuberculous peritonitis, and twisted ovarian tumours. 3. The symptoms arise from acute pancreatitis. 4. The intestine is extensively paralysed by embolism or thrombosis of the mesenteric vessels. 5. The constipation, vomiting, and perhaps distension are due to toxic causes such as lead-poisoning and uræmia. 6. The symptoms of intestinal obstruction are nervous in origin, and arise from functional derangements of the nervo-muscular mechanism of the alimentary canal, as in gastric crises, and constipation in tabes and other obscure nervous conditions. 7. The bowels are not obstructed, but the patient vomits and is very constipated, and there is sometimes a visible peristalting viscus or a tumour in the abdomen, as in pyloric obstruction, or cancer of the greater curvature of the stomach. Barnard[2] affirms that in the great majority of cases the diagnosis of intestinal obstruction is easy, and that the most important sign of all is that the second of two turpentine enemata is returned with little force and without fæces or flatus. A case showing the occasional value of Nélaton's operation (enterostomy) for intestinal obstruction is recorded by Newbolt.[3]

[1] Clin. Jour., June 14, 1905, p. 129, and July 5, 1905, p. 183. [2] Ibid., July 12, 1905, p. 206. [3] Lancet, September 9, 1905, p. 763.

